# Restoration of mutant K-Ras repressed miR-199b inhibits K-Ras mutant non-small cell lung cancer progression

**DOI:** 10.1186/s13046-019-1170-7

**Published:** 2019-04-15

**Authors:** Hua Jin, Yoonjeong Jang, Nian Cheng, Qing Li, Peng-Fei Cui, Zhi-Wei Zhou, Hu-Lin Jiang, Myung-Haing Cho, Kenneth D. Westover, Qun-You Tan, Cheng-Xiong Xu

**Affiliations:** 1 Department of Thoracic Surgery, Daping Hospital and Research Institute of Surgery, Third Military Medical University, Chongqing, 400042 China; 2 Laboratory of Toxicology, College of Veterinary Medicine, Seoul National University, Seoul, 151-742 South Korea; 3 Cancer Center, Daping Hospital and Research Institute of Surgery, Third Military Medical University, Chongqing, 400042 China; 4 State Key Laboratory of Natural Medicines, Department of Pharmaceutics, China Pharmaceutical University, Nanjing, 210009 China; 5 Department of Biochemistry and Radiation Oncology, The University of Texas Southwestern Medical Center, Dallas, TX 75390 USA

**Keywords:** miR-199b, K-Ras, Non-small cell lung cancer

## Abstract

**Background:**

miRNAs play crucial role in the progression of K-Ras-mutated nonsmall cell lung cancer (NSCLC). However, most studies have focused on miRNAs that target K-Ras. Here, we investigated miRNAs regulated by mutant K-Ras and their functions.

**Methods:**

miRNAs regulated by mutant K-Ras were screened using miRNA arrays. miR-199b expression levels were measured by qRT-PCR. The protein expression levels were measured using Western blot and immunohistochemistry. The effects of miR-199b on NSCLC were examined both in vitro and in vivo by overexpressing or inhibiting miR-199b. DNA methylation was measured by bisulfite sequencing.

**Results:**

An inverse correlation was observed between K-Ras mutation status and miR-199b levels in NSCLC specimens and cell lines. The inhibition of miR-199b stimulated NSCLC growth and metastasis, while restoration of miR-199b suppressed K-Ras mutation-driven lung tumorigenesis as well as K-Ras-mutated NSCLC growth and metastasis. miR-199b inactivated ERK and Akt pathways by targeting K-Ras, KSR2, PIK3R1, Akt1, and Rheb1. Furthermore, we determined that mutant K-Ras inhibits miR-199b expression by increasing miR-199b promoter methylation.

**Conclusion:**

Our findings suggest that mutant K-Ras plays an oncogenic role through downregulating miR-199b in NSCLC and that overexpression of miR-199b is a novel strategy for the treatment of K-Ras-mutated NSCLC.

**Electronic supplementary material:**

The online version of this article (10.1186/s13046-019-1170-7) contains supplementary material, which is available to authorized users.

## Background

Lung cancer is the leading cause of cancer-related death worldwide; moreover, nonsmall-cell lung cancer (NSCLC) is the predominant type, as it accounts for 80% of all lung cancers [1]. The initiation of lung cancer is caused by a number of factors, including genetic alterations such as mutations in K-Ras, epidermal growth factor receptor (EGFR), anaplastic lymphoma kinase (ALK), phosphoinositide 3-kinase (PI3K), and BRAF [2–4]. Among these, K-Ras is the most frequently mutated oncogene found in NSCLC, 29% NSCLC patients have K-Ras mutation [5]. Notably, K-Ras mutations are not only related to cancer initiation; they also promote cancer progression, like metastasis [6] and therapeutic resistance [7] Thus, K-Ras is a high-priority therapeutic target, but despite the decades of effort, no targeted therapy is clinically available for K-Ras-mutated cancers [8].

MicroRNAs (miRNAs) are ~ 20–23 nucleotide single-stranded small noncoding RNAs that negatively regulate target gene expression by promoting the degradation or inhibiting the translation of the mRNA of target genes. Dysregulated expression of miRNAs is found in most types of cancers, and these miRNAs can promote cancer initiation and progression [9]. Interestingly, miRNA dysregulation has been linked to the activation of certain oncogenes, such as K-Ras mutations [10]. Recent studies showed that overexpression or inhibition of certain miRNAs can significantly suppress the progression of K-Ras-mutated NSCLC by targeting K-Ras [1, 8], suggesting that artificial modulation of certain miRNAs that are associated with K-Ras-driven cancers may be a useful therapeutic strategy for treating K-Ras-mutated NSCLC. However, most studies have focused on finding miRNAs that can target K-Ras.

Here, we report that level of miR-199b was inversely correlated with K-Ras mutations in NSCLC, and mutant K-Ras inhibits miR-199b expression by increasing miR-199b promoter methylation. Importantly, restoration of miR-199b inhibited K-Ras-mutated NSCLC growth, metastasis and K-Ras mutation-driven lung tumorigenesis through the inhibition of Akt and ERK signaling by directly targeting K-Ras and multiple coactivators of Akt and ERK signaling. Our results suggest that K-Ras mutations cause lung tumorigenesis and progression partly through the inhibition of miR-199b expression and that restoring miR-199b expression may be a useful strategy for the treatment of K-Ras-mutated NSCLC.

## Materials and methods

### Materials

In Situ Cell Death Detection Kit, 5-azacytidinecytidine, anti-caspase 3 antibody, anti-actin antibody, anti-p70S6K (Thr389), fetal bovine serum (FBS) and cell culture medium were obtained from Sigma (St. Louis, MO). Dual-Luciferase Assay Kit and Invasion Assay Kit were purchased from Promega (Madison, WI) and BD Biosciences (San Jose, CA), respectively. TRIzol, cDNA reverse transcription kit, miRNA luciferase reporter vector, primer sets for miR-199b and U6, Lipofectamine 2000, SYBR Green PCR kit, miR-199b mimics, control oligonucleotides, and miRNA assay kit were purchased from Life Technologies (Carlsbad, CA). Human miRNA Array v2.0 and Human genome U133 Plus 2 array were obtained from Arraystar (Rockville, MD) and Affymetrix (Santa Clara, CA), respectively. shRNA of K-Ras, Renilla Luciferase, and K-Ras (G12D) expression constructs were kindly provided by Dr. Cheng (Moffitt Cancer Center). Antibodies against K-Ras, KSR2, PIK3R1, Rheb1, Akt1, phospho-Akt (Ser473), phospho-ERK (Thr202/Tyr204), phospho-mTOR (Ser2448), and Ki-67 were purchased from Abcam (Cambridge, MA). K-Ras (G12D) antibody was obtained from Cell Signaling Technology, Inc.

### Cell culture and specimens

H157, H1975, H2172, HCC827, H2122, H441, A549 and H460 were purchased from American Type Culture Collection (Manassas, VA). PC-9 and H125 were kindly provided by Dr. Shen (Jilin University, China). All cell lines were cultured in Dulbecco’s Modified Eagle Medium supplemented with 10% FBS. NSCLC specimens were collected before treatment from patients with newly diagnosed NSCLC at Daping Hospital, Third Military Medical University. This experiment was approved by the ethical review committees of Daping Hospital, Third Military Medical University.

### RNA isolation and analysis

Total RNA was isolated from tissues and cells using TRIzol according to the manufacturer’s instructions. miR-199b and U6 were analyzed using a TaqMan miRNA Assay Kit. The relative expression of miR-199b was normalized against U6 expression using the 2^-△Ct^ method, and the miR-199b expression fold-change in NSCLC tissue was matched to nontumor control samples for evaluation. For other genes, RT and PCR were performed with the cDNA RT Kit and SYBR Green PCR Kit, respectively. Primer sequences are listed in Additional file 1: Table S1. miRNAs affected by K-Ras were detected using Human miRNA Array v2.0 and genes affected by miR-199b were detected using Human genome U133 Plus 2 array.

### Luciferase reporter assay

The 3`-UTR segments of genes that were predicted to interact with miR-199b were amplified by PCR from human genomic DNA and inserted into *Mlu*Iand *Hind*III sites of the miRNA Expression Reporter Vector. Luciferase assay was performed using HEK293 cells as previously described [11].

### Immunoblotting

Western blotting and immunohistochemistry were performed as previously described [12]. Western blot band intensity was quantified using Image J software (National Institutes of Health, Bethesda, MD).

### Invasion, cell viability and cell proliferation assay

Cells were transfected with the indicated oligonucleotides for 24 h and then subjected to analysis. Invasion, cell viability and cell proliferation assays were performed as previously described [11].

### Colony formation assay

After 24 h of oligonucleotide transfection, cells were trypsinized and resuspended in 0.5 ml 0.35% agar in growth medium at a density of 2500 cells/well (6-well plate). Then, the agar-cell mixture was plated on the top of a solid layer of 0.8% agar in growth medium. Colonies were counted 12 days later.

### Animal experiments

miR-199b effects on K-Ras mutation-driven lung tumorigenesis were examined using 6-week-old female K-Ras^LA1^ transgenic mice at Seoul National University, Korea. miR-199b expression plasmids or empty vectors were mixed with the gene delivery nanoparticle, PCA_m_H_n_, as previously described [13], and this mixture was then delivered to mice using an aerosol-based, nose-only exposure chamber system as previously described [14]. Briefly, mice were randomly divided into three treatment groups (6 mice per group), and then mice were exposed to aerosol containing 10 mg of PCA_m_H_n_ with or without 1 mg plasmid (miR-199b plasmid or empty vector). K-Ras^LA1^ mice were exposed to aerosol twice a week for one month.

For the subcutaneous tumor growth assay, 2 × 10^6^ indicated cells in 0.1 ml of phosphate-buffered saline were subcutaneously injected into 6-week-old male nude mice. One month after cell injection, these mice were sacrificed.

For lung metastasis experiments, 5 × 10^5^ indicated cells were suspended in 0.1 ml of PBS and injected into the lateral tail vein of 6-week-old male nude mice. One month after injection, mice were sacrificed, and lung surface tumor foci were counted. This experiment was conducted at the Daping Hospital and Research Institute of Surgery, Third Military Medical University. All animal experiments were approved by the Animal Care and Use Committees of the appropriate institutions.

### DNA methylation assay

Genomic DNA was isolated using the QIAGEN DNA extraction kit, and 1 μg of genomic DNA was treated with sodium bisulfite. The bisulfite-treated DNA was desalted and eluted in 40 μl of elution buffer; then, 2 μl of DNA was amplified with the forward primer 5′-TTAAAGAGGTTGGGTATGAG-3′ and the reverse primer 5′-ATCCTCTAATCCATCCAAAC-3′. PCR products were ligated into the TA cloning vector, and the DNA sequences were determined using the following primers: forward primer: 5′-GGAGGAGAGGAGGAAGTT-3′; reverse primer: 5′-CCAACCTATATCCCCCTAAC-3′.

### Statistical analysis

All data are presented as the mean ± standard deviation, and significant differences between treatment groups were analyzed by Student’s t-test or one-way analysis of variance (ANOVA) and Duncan’s multiple range test using SAS statistical software version 6.12 (SAS Institute). Differences were considered statistically significant at a *p* value of less than 0.05.

## Results

### miR-199b expression was negatively regulated by mutant K-Ras in NSCLC

**Fig. 1:**
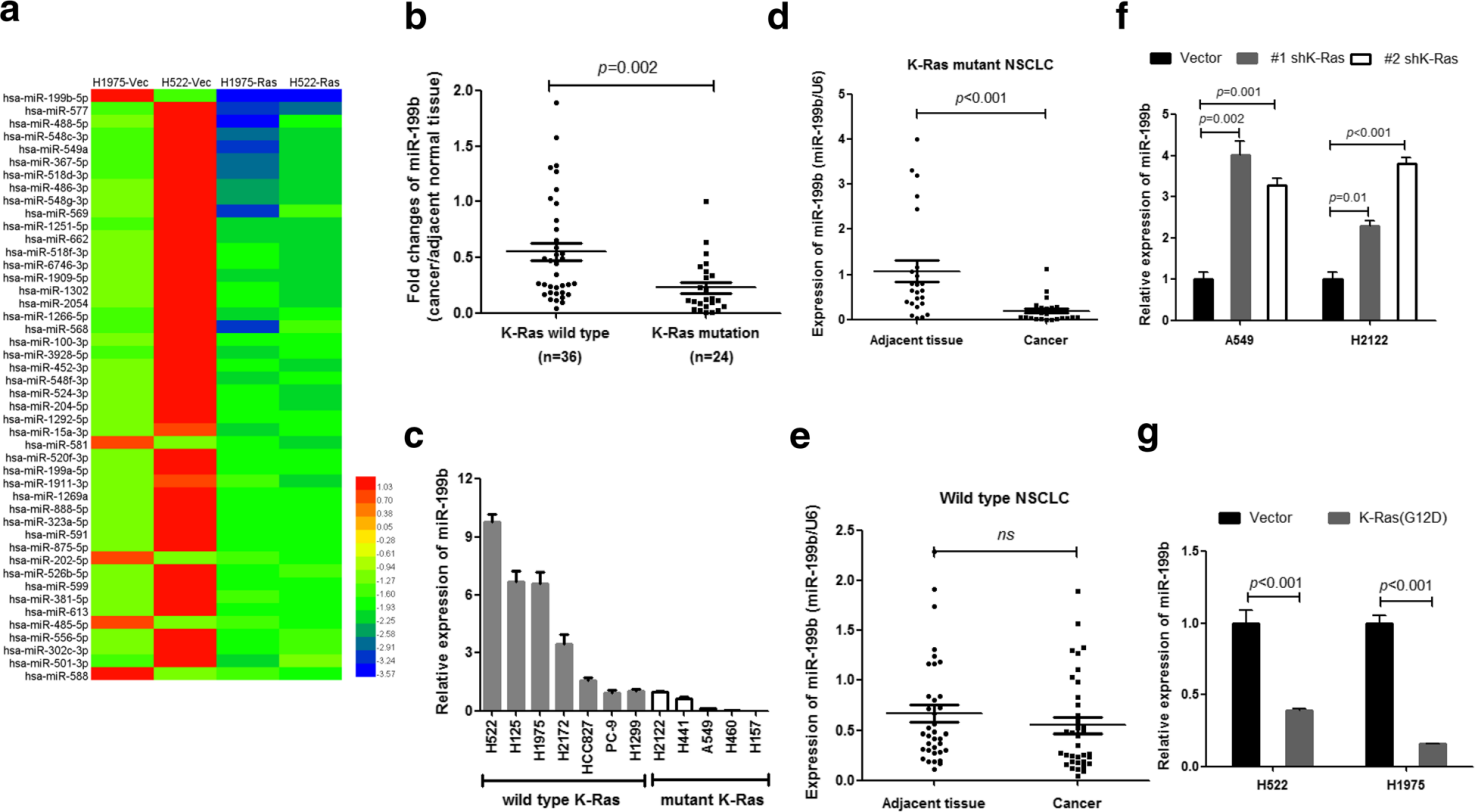
Mutant K-Ras suppresses miR-199b expression in NSCLC. **a** Heatmap showing the 46 significantly downregulated miRNAs in K-Ras (G12D)-overexpressing NSCLC cell lines. **b** miR-199b expression is downregulated in specimens from NSCLC patients with K-Ras mutations (*n* = 24) compared to those from K-Ras wild-type specimens (*n* = 36). **c** K-Ras-mutated NSCLC cell lines have lower miR-199b expression levels than NSCLC cell lines with wild-type K-Ras. **d** miR-199b was significantly decreased in K-Ras-mutated NSCLC specimens compared to their respective adjacent tissues (*n* = 24). **e** No difference was observed between adjacent tissues and NSCLC tissue from K-Ras wild-type patients (*n* = 36). **f** Silencing of K-Ras increased miR-199b expression in both K-Ras-mutated NSCLC cell lines, A549 and H2122. Cells were transfected with a K-Ras shRNA expression vector or a scrambled vector for 72 h and then subjected to miR-199b analysis. **g** Overexpression of mutant K-Ras (G12D) inhibits miR-199b expression in both K-Ras wild-type NSCLC cell lines, H522 and H1975. Cells were transfected with a K-Ras (G12D) expression vector or an empty vector for 72 h and then subjected to miR-199b analysis

To identify miRNAs that are regulated by mutant K-Ras in NSCLC, we performed miRNA array assays using the K-Ras (G12D)-overexpressing NSCLC cell lines H1975 and H522, as well as their vector control cells. As shown in Fig. 1a, we detected a total of 46 miRNAs that were significantly decreased by K-Ras (G12D) overexpression in both NSCLC cell lines compared to the respective control cells. Among them, miR-199b was the most significantly inhibited. Consistent with miRNA array results, clinical samples and NSCLC cell line analysis results showed that miR-199b was dramatically decreased in K-Ras-mutated NSCLC specimens and cell lines compared to wild- type K-Ras NSCLC specimens and cell lines (Fig. 1b and c). Additionally, we demonstrated decreased expression of miR-199b in K-Ras-mutated NSCLC specimens compared to their adjacent tissues (Fig. 1d). However, we did not observe differences between adjacent tissues and tumors in K-Ras wild-type NSCLC patients (Fig. 1e). These data strongly suggested that mutant K-Ras may be involved in the negative regulation of miR-199b in NSCLC. To test this hypothesis, we measured the expression of miR-199b after knockdown or overexpression of mutant K-Ras (G12D) (Additional file 1: Figure S1) in NSCLC cells with mutant or wild-type K-Ras, respectively. Our data show that knockdown of K-Ras significantly increased miR-199b expression, while overexpression of mutant K-Ras suppressed miR-199b expression (Fig. 1f and g). Taken together, our results support the idea that mutant K-Ras suppresses the expression of miR-199b in NSCLC.

### Inhibition of miR-199b significantly contributes to NSCLC progression

**Fig. 2:**
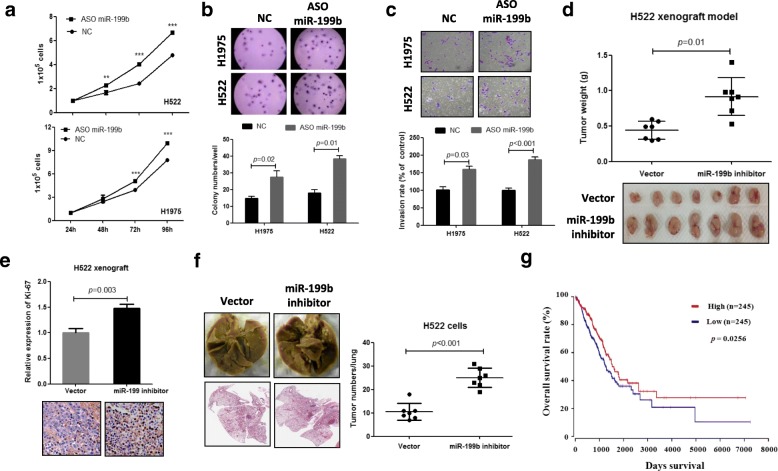
Inhibition of miR-199b stimulates NSCLC progression. **a-c** Inhibition of miR-199b promotes cell proliferation, soft agar colony formation, and cell invasion in both H522 and H1975 NSCLC cell lines. Cells were transfected with negative control oligonucleotides (NC) or antisense oligonucleotides of miR-199b (ASO miR-199b) and were then subjected to cell proliferation, soft agar colony formation and invasion analysis. **d-e** Inhibition of miR-199b stimulates tumor growth and cancer cell proliferation in the H522 xenograft model. Using stably expressing miR-199b-antisense H522 cells or control cells, xenografts were generated in nude mice. One month after cell injection, mice were sacrificed, and the tumor weights were measured. Cell proliferation was measured using Ki-67 immunohistochemistry in tumor tissues. **f** Inhibition of miR-199b promotes NSCLC cell metastasis in vivo. Stably expressing miR-199b-antisense H522 cells or control cells were injected into nude mice by tail vein injection. The mice were sacrificed 1 month after cell injection, and the tumors on the lung were counted. **g** Kaplan-Meier analysis of the overall survival of patients with lung adenocarcinoma for whom overall survival information was available in the TCGA lung cancer data set

To further validate the effects of downregulated miR-199b expression on NSCLC progression, we performed cell-based experiments where we expressed antisense oligonucleotides in the high-expressing miR-199b NSCLC cell lines H522 and H1975. As expected, inhibition of miR-199b (Additional file 1: Figure S2a) strongly promoted cell proliferation (Fig. 2a), soft agar colony formation (Fig. 2b) and invasion (Fig. 2c) in both H522 and H1975 cells. To further confirm these in vitro experimental results in vivo, nude mice were subcutaneously inoculated with miR-199b-silenced H522 cells and the corresponding control cells. Consistent with the in vitro results, animal experiments showed that inhibition of miR-199b (Additional file 1: Figure S2b) significantly stimulated tumor growth (Fig. 2d) and cancer cell proliferation (Fig. 2e) compared to the vector control.

We also investigated the effects of downregulated miR-199b on lung cancer metastasis by tail vein injection of H522 cells that stably express miR-199b-antisense oligonucleotides into nude mice, and we assessed tumor nodule burden after 1 month. Our data show that inhibition of miR-199b dramatically increased NSCLC cell lung metastasis (Fig. 2f). These in vivo and in vitro data suggest that miR-199b may affects clinical outcome of NSCLC patients. In fact, TCGA dataset analysis showed that decreased miR-199b expression levels were significantly correlated with poor survival rates in patients with lung adenocarcinoma (Fig. 2g). Taken together, these findings support a model where inhibition of miR-199b significantly contributes to NSCLC progression.

### Restoring miR-199b inhibits K-Ras-mutated NSCLC progression and K-Ras mutation-driven lung tumorigenesis

**Fig. 3:**
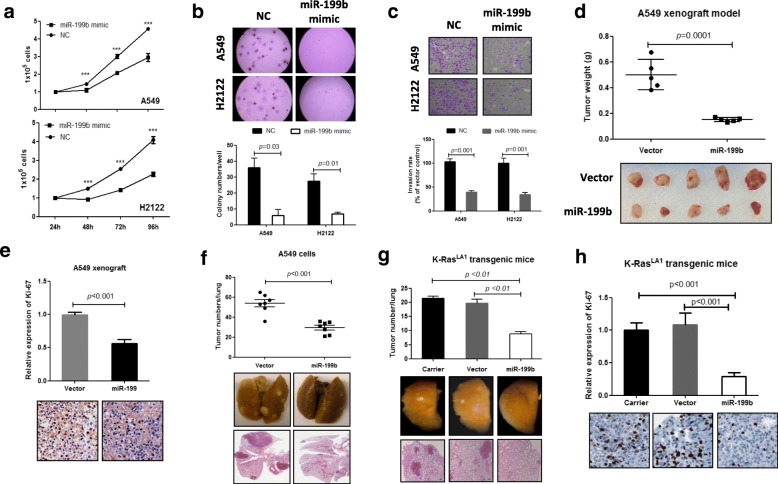
Overexpression of miR-199b dramatically inhibits K-Ras mutation-driven lung tumorigenesis and progression. **a-c** Overexpression of miR-199b inhibited cell proliferation, soft agar colony formation and cell invasion in both K-Ras-mutated NSCLC cell lines, A549 and H2122. Cells were transfected with negative control oligonucleotides (NC) or miR-199b mimics and then subjected to cell proliferation, soft agar colony formation and invasion assays. **d-e** Overexpression of miR-199b inhibited tumor growth and cancer cell proliferation in the A549 xenograft models. Stably expressing miR-199b A549 and vector control cells were used to generate a xenograft model in nude mice. Tumors were collected 1 month after cell injection, and the tumor weights were measured. Cell proliferation was analyzed using Ki-67 IHC in tumor tissues. **f** Overexpression of miR-199b inhibited NSCLC cell metastasis in vivo. Stably expressing miR-199b A549 or control cells were injected into nude mice by tail vein injection. The mice were sacrificed 1 month after cell injection, and the tumors on the lung surface were counted. **g** Aerosol delivery of miR-199b to the lungs inhibits K-Ras mutation-driven lung tumorigenesis. K-Ras^LA1^ transgenic mice were exposed to a mixture of miR-199b expression plasmid with the gene carrier (miR-199), gene carrier only (Carrier) or empty plasmid only (vector) using a nose-only aerosol delivery system. Four weeks after gene delivery, mice were sacrificed, and the number of tumor nodules on the lung surface were counted. **h** Aerosol delivery of miR-199b inhibited cancer cell proliferation in lung tumor of K-Ras^LA1^ transgenic mice. The Ki-67 expression was detected using immunohistochemistry

Given that the K-Ras mutation inhibits the expression of miR-199b and that the inhibition of miR-199b promotes NSCLC progression, we hypothesized that restoring miR-199b could suppress K-Ras-mutated NSCLC. Cell-based assays on the K-Ras-mutated NSCLC cell lines A549 and H2122 with overexpressed miR-199b (Additional file 1: Figure S2c) showed significant suppression of cell proliferation (Fig. 3a), soft agar colony formation (Fig. 3b) and invasion (Fig. 3c) in both cell lines. Then, we tested the effects of miR-199b overexpression on K-Ras-mutated NSCLC growth in xenograft models that were generated by stably expressing miR-199b in A549 cells (Additional file 1: Figure S2d). We found that overexpression of miR-199b significantly inhibited tumor growth (Fig. 3d) and cancer cell proliferation (Fig. 3e) compared to the vector control. We also confirmed the in vivo anti-metastatic effects of miR-199b using a lung metastasis model that was generated by stably expressing miR-199b in A549 cells. As shown in (Fig. 3f), miR-199b-overexpressing A549 cells formed fewer tumor nodules in the lung than the vector control, demonstrating that miR-199b can inhibit the metastasis of K-Ras-mutated NSCLC cells.

Next, we investigated the effects of miR-199b on K-Ras mutation-driven lung tumorigenesis. To verify the effects of miR-199b on K-Ras mutation-driven lung tumorigenesis, we used aerosol-based, nose-only exposure methods to deliver miR-199b to the lungs of K-Ras^LA1^ transgenic mice. To enhance the aerosol gene delivery efficiency, we used the gene carrier particle PCA_m_H_n._ As shown in Additional file 1: Figure S3a, we delivered a green fluorescent protein (GFP) expression plasmid with the gene carrier, which significantly enhanced the GFP signal compared with plasmid-only controls, suggesting that PCA_m_H_n_ is an efficient gene delivery carrier. In fact, miR-199b expression plasmid delivery with the gene carrier PCA_m_H_n_ significantly increased miR-199b levels in the lung tissues of K-Ras^LA1^ transgenic mice compared to the carrier and vector control groups (Additional file 1: Figure S3b). Notably, such delivery of miR-199b to the lungs of K-Ras^LA1^ transgenic mice dramatically decreased the numbers of tumors in the lung (Fig. 3g). Histopathologic examination also indicated that pulmonary tumor formation was significantly suppressed by miR-199b delivery (Fig. 3g). In addition, miR-199b delivery significantly inhibited the expression of the cell proliferation marker protein Ki-67 compared to other groups (Fig. 3h). Taken together, restoration of miR-199b can suppress K-Ras mutation-driven lung tumorigenesis, and restoration of miR-199b may be a useful strategy for treating K-Ras-mutated NSCLC.

### miR-199b targets K-Ras and multiple genes that are involved in both Akt and ERK signaling activation

**Fig. 4:**
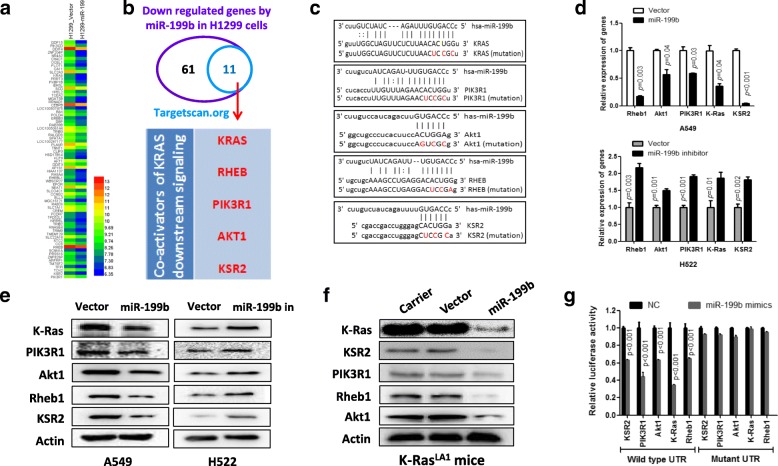
miR-199b targets several components of the RAS-ERK and PI3K/Akt signaling pathways. **a** Heatmap showing the 72 downregulated genes in miR-199b-overexpressing H1299 cells. **b** Venn diagram showing candidate target genes of miR-199b in NSCLC. Candidate target genes of miR-199b were indicated using TargetScanHuman from the 72 genes that were downregulated by miR-199b overexpression in H1299 cells. **c** Sequence alignment of miR-199b with the 3`-UTR of the K-Ras, PIK3R1 (PI3K p85), Akt1, Rheb and KSR2 genes. **d-e** miR-199b negatively regulates the expression of the candidate target genes of miR-199b at both the mRNA and protein levels in NSCLC cells. The mRNA and protein expression levels of the indicated genes were measured using qRT-PCR and Western blot analysis in stably expressing miR-199b A549 cells and stably expressing miR-199b-antisense H522 cells. **f** The expression of candidate target genes of miR-199b was significantly decreased in lung tissues from miR-199b-delivered K-Ras^LA1^ transgenic mice. The expression levels of the indicated proteins were detected in lung tissues of miR-199b-delivered K-Ras^LA1^ transgenic mice and their control groups using Western blot analysis. **g** 3`-UTR luciferase reporter assay for candidate genes of miR-199b. HEK293 cells were cotransfected with miRNA luciferase reporter plasmid harboring indicated gene mutants or wild-type 3’UTR and miR-199b mimics or negative oligonucleotides control (NC). After 48 h of transfection, luciferase intensity was assessed

To investigate the anticancer mechanism of miR-199b, we performed gene array analysis using miR-199b-overexpressing H1299 cells and their control cells. As shown in Fig. 4a, we detected a total 72 genes that were downregulated more than 1.5-fold in miR-199b-overexpressing H1299 cells compared to control cells. Among them, we found that 11 genes contained putative miR-199b target sites in their 3`-UTRs (www.targetscan.org) (Fig. 4b). Interestingly, 5 of the genes are involved in the activation of the K-Ras downstream signaling pathway, including K-Ras, Akt1, Rheb1, PIK3R1 and KSR2 (Fig. 4c). To test whether miR-199b regulates the expression of candidate target genes, we examined the expression levels of these targets in stably expressing miR-199b or miR-199b-antisense NSCLC cells. Our results show that miR-199b negatively regulated the expression of all candidate genes, as indicated by both the mRNA (Fig. 4d) and protein levels (Fig. 4e and Additional file 1: Figure S4a). We also observed downregulation of miR-199b candidate target genes in the lung tissues of K-Ras^LA1^ transgenic mice in which miR-199b was delivered compared to the lungs of control mice (Figs. 4f and Additional file 1: Figure S4b). Furthermore, we demonstrated a direct interaction between miR-199b and the 3′-UTR of target genes using a miRNA luciferase reporter assay. We expressed luciferase constructs containing the 3′-UTR of miR-199b target genes. In all cases, luciferase activity was reduced in the presence of miR-199b mimics (Fig. 4g). In contrast, luciferase activity was unchanged in negative controls in which putative seed sequences were mutated (Fig. 4g).

**Fig. 5:**
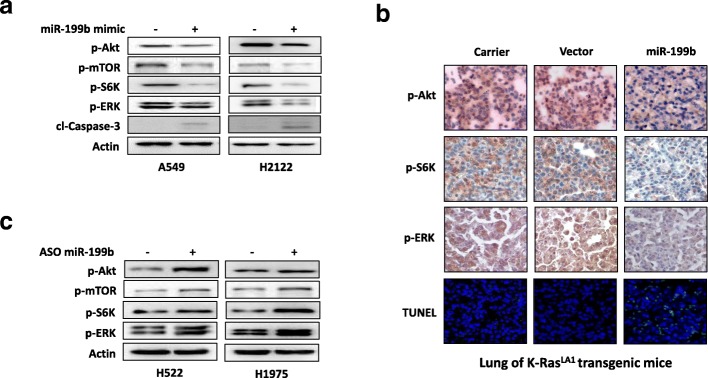
Overexpression of miR-199b simultaneously suppresses the activation of Akt and ERK signaling pathways both in vitro and in vivo. **a** Overexpression of miR-199b inhibited Akt and ERK signaling and stimulated apoptosis in A549 and H2122 NSCLC cell lines with K-Ras mutations. The indicated cells were transfected with negative oligonucleotide controls or miR-199b mimics. After 72 h of transfection, cells were subjected to Western blot analysis. **b** Aerosol delivery of miR-199b inhibited the Akt and ERK signaling pathways and stimulated apoptosis in lung tumors of K-Ras^LA1^ transgenic mice. The indicated proteins were detected using immunohistochemistry, and the apoptotic cells were detected using TUNEL assay. **c** Inhibition of miR-199b activated Akt and ERK signaling. The indicated cells were transfected with negative oligonucleotide controls (NC) or antisense nucleotides of miR-199b. After 72 h of transfection, cells were subjected to Western blot analysis

We then tested whether miR-199b negatively regulates Akt and ERK signaling in NSCLC. As shown in Fig. 5a and Additional file 1: Figure S5a, overexpression of miR-199b significantly inhibited the phosphorylation of Akt, mTOR, S6K and ERK in NSCLC cell lines, and cleaved caspase-3 levels increased. Consistent with the in vitro results, K-Ras^LA1^ transgenic mice experiments also showed that delivery miR-199b can significantly inhibit ERK and Akt signaling activation and induce cancer cell apoptosis (Fig. 5b). In contrast, inhibition of miR-199b significantly stimulated Akt and ERK signaling in NSCLC cells (Figs. 5c and Additional file 1: Figure S5b). Taken together, our findings indicated that miR-199b inhibited Akt and ERK signaling by directly targeting K-Ras and multiple coactivators of Akt and ERK signaling in NSCLC.

### Mutant K-Ras inhibits miR-199b expression by increasing miR-199b promoter DNA methylation

**Fig. 6:**
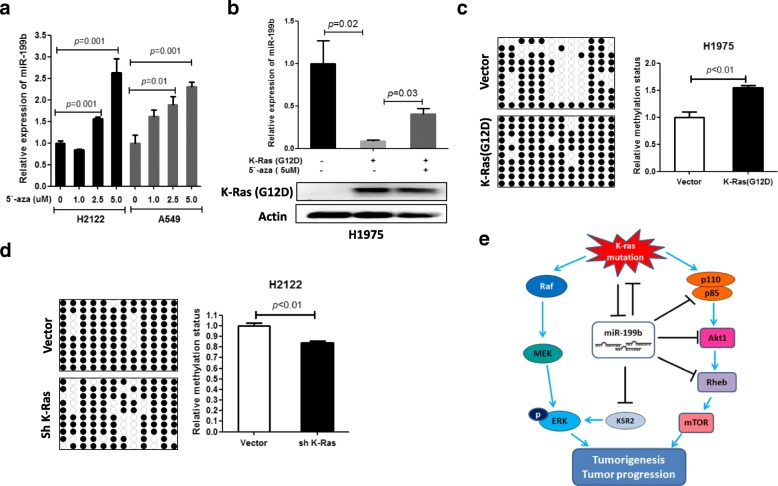
Mutant K-Ras inhibits miR-199b expression in NSCLC cells by stimulating DNA methylation of the miR-199b promoter. **a** Demethylation agent treatment increased miR-199b expression in K-Ras-mutated NSCLC cell lines. H2122 and A549 cells were treated with the indicated concentration of 5-azacytidine (5`-aza) for 12 days. Next, they were subjected to miR-199b expression analysis. Cell culture medium was changed every third day. **b** Demethylation agent treatment abolished mutant K-Ras overexpression-induced inhibition of miR-199b expression. H1975 cells were transfected with mutant K-Ras (G12D) expression plasmid or empty vectors. After 24 h of transfection, cells were treated with or without 5 μM 5`-aza for 6 days. Then, cells were subjected to qRT-PCR and Western blot analysis. **c** Mutant K-Ras increased DNA methylation of the miR-199b promoter in H1975 cells. Cells were transfected with K-Ras (G12D). After 96 h of transfection, cells were subjected to bisulfite sequencing analysis. **d** Silencing of K-Ras inhibits DNA methylation of the miR-199b promoter in the K-Ras mutated cell H2122. Cells were transfected with K-Ras shRNA expression vector or scramble vector. After 96 h of transfection, cells were subjected to bisulfite sequencing analysis. **e** A schematic model of lung tumorigenesis regulation and the progression of K-Ras-mutated NSCLC by the miR-199b regulatory axis

Previous studies showed that K-Ras can regulate gene expression by altering DNA methylation [15] and that the expression level of miR-199b is determined by its promoter methylation status [16]. We therefore suspected that mutant K-Ras-dependent suppression of miR-199b expression in NSCLC cells might be due to increased methylation in the miR-199b promoter. As expected, miR-199b expression levels were restored after treatment with the DNA methylation inhibitor 5-azacytidinecytidine in NSCLC cells containing K-Ras mutations (Fig. 6a). Consistent with these results, mutant K-Ras-dependent downregulation of miR-199b was blocked by 5-azacytidinecytidine treatment in wild-type K-Ras NSCLC cells (Fig. 6b). Moreover, bisulfite sequencing and quantitative methylation-specific PCR of the miR-199b CpG island region confirmed that methylation levels of the miR-199b promoter are increased in NSCLC cells overexpressing mutant K-Ras (Fig. 6c). In contrast, the silencing of mutant K-Ras decreased methylation levels (Fig. 6d). These findings indicated that mutant K-Ras inhibits miR-199b expression by increasing the methylation of the miR-199b promoter.

## Discussion

Downregulated expression of miR-199b has been identified in several types of tumors, including hepatocellular carcinoma [17], leukemia [18], glioma [19], ovarian cancer [16], colorectal cancer and breast cancer [20, 21]. Dysregulated expression of miR-199b was also detected in lung cancer previously by several research groups, but these results are inconsistent. For example, Kim et al. [22] reported that miR-199b was upregulated in lung adenocarcinoma, but Wang et al. [23] reported that miR-199b was downregulated in NSCLC. In addition, the regulatory mechanism of miR-199b in NSCLC is not clear. In this study, we found that the expression level of miR-199b was inversely correlated with K-Ras mutations in human NSCLC specimens and NSCLC cell lines. In addition, in vitro data show that miR-199b expression was inhibited by overexpression of mutant K-Ras and increased by silencing of mutant K-Ras in NSCLC cells, suggesting that mutant K-Ras negatively regulates miR-199b expression in NSCLC. This is the first study to show direct evidence that downregulated expression levels of miR-199b are caused by K-Ras mutation in NSCLC.

Next, we elucidated the mechanism by which mutant K-Ras suppresses miR-199b expression. A previous study showed that promoter methylation suppresses the expression of miR-199b in solid cancers [24]. Here, we showed that demethylation drug treatment causes restoration of miR-199b expression in K-Ras mutant NSCLC cells, and mutant K-Ras-dependent inhibition of miR-199b expression was abolished by demethylation drug treatment in NSCLC cells, suggesting that decreased expression of miR-199b in K-Ras mutant NSCLC cells was associated with the methylation status of the miR-199b promoter. In addition, our data showed that overexpression of mutant K-Ras increased the methylation of the miR-199b promoter in NSCLC cells, whereas silencing of mutant K-Ras decreased the methylation of the miR-199b promoter. These findings strongly suggest that mutant K-Ras suppresses miR-199b expression by increasing DNA methylation at the miR-199b promoter in NSCLC cells. However, the detailed mechanism of how mutant K-Ras regulates the methylation of the miR-199b promoter will need to be studied in the future.

Here, we also provide multiple evidences for the role of miR-199b in regulating K-Ras-driven lung tumorigenesis and progression. K-Ras-mutant cancers are among the most difficult to treat; they are associated with poor patient survival [25]. Our data show that mutant K-Ras suppresses miR-199b expression and that inhibition of miR-199b stimulates NSCLC growth and metastasis. Congruently, miR-199b restoration dramatically suppressed K-Ras mutation-driven lung tumorigenesis, as well as K-Ras-mutated NSCLC growth and metastasis. These findings indicate that K-Ras mutant-mediated tumorigenesis and progression are partly dependent on the inhibition of miR-199b expression in the lungs. Additionally, these findings indicate that restoration of miR-199b is a useful strategy for treating K-Ras-mutated NSCLC. This study is the first report to show that decreased expression of miR-199b is involved in K-Ras mutation-driven lung tumorigenesis and progression.

Furthermore, we evaluated the anticancer mechanism of miR-199b. Attempts to therapeutically target members of RAS pathways, such as PI3K/Akt, RAF, MEK and ERK, have yielded mixed results. This has led to the current wave of multiagent trials (REFS) [8]. Evidence shows that simultaneous inactivation of either of the two pathways could be a strategy for the treatment of K-Ras-mutated NSCLC. For example, Engelman et al. reported that treatment of K-Ras-mutant mice with a single inhibitor of the Akt or ERK pathway led to only modest tumor regression. However, combination treatment of Akt and ERK signaling inhibitors induced markedly synergistic tumor regression [26]. miR-199b significantly inhibits both PI3K/Akt and ERK signaling activation in vitro and in vivo, suggesting that miR-199b exerts its anticancer function through the simultaneous inhibition of both Akt and ERK signaling in NSCLC.

Finally, we elucidated the mechanism by which miR-199b inhibits the Akt and ERK signaling pathways in NSCLC cells. Recent studies have shown that one single miRNA plays its role through strongly influencing a specific signaling pathway by simultaneously targeting multiple components of that specific signaling pathway. For example, Fang et al. reported that increased miR-582-3p expression induces lung cancer recurrence through activating Wnt/β-catenin signaling by simultaneously inhibiting multiple inhibitors of Wnt/β-catenin signaling, such as AXIN2, DKK3 and SFRP1 [27]. Lin et al. reported that miR-135b activates the hippo pathway by targeting multiple key components of the pathway, including LATS2, NDR2 and LTS1, to significantly stimulate lung cancer metastasis [28]. Here, we identified a series of novel target genes of miR-199b that belong to PI3K/Akt and ERK signaling pathways, including K-Ras, PIK3R1, Akt1, Rheb1 and KSR2. K-Ras is an upstream regulator of PI3K/Akt and ERK signaling, and mutations in K-Ras lead to the activation of these two oncogenic pathways in NSCLC [29]. PIK3R1, Akt1, and Rheb1 have been identified as important components of PI3K/Akt signaling activation, and they have been suggested as therapeutic targets in lung cancer treatment [12, 30, 31]. Finally, KSR2 is an activator of ERK signaling [32–34], and its overexpression stimulates tumor cell transformation [35]. These findings suggest that miR-199b inhibits the PI3K/Akt and ERK pathways by simultaneously inhibiting a number of their activators.

## Conclusion

In conclusion, our findings suggest that mutant K-Ras stimulates lung tumorigenesis and progression partly through the inhibition of miR-199b expression by promoting miR-199b promoter methylation. Restoration of miR-199b may be a useful strategy for inhibiting K-Ras mutation-driven lung tumorigenesis and for treating K-Ras-mutated NSCLC. miR-199b plays an anticancer role by simultaneously inhibiting the Akt and ERK pathways by directly targeting several components of the Akt and ERK pathways in NSCLC (Fig. 6e).

## Additional file

Additional file 1: **Table S1** Primer sequences used for qRT-PCR. **Figure S1** Expression of K-Ras in NSCLC cells that overexpressed or silenced K-Ras. **a** shRNA of K-Ras significantly suppressed K-Ras expression in NSCLC cells. Indicated cells were transfected with indicated shRNA of K-Ras. After 72 hours of transfection, cells were subjected to Western blot analysis. **b** Transfection of K-Ras (G12D) expression vector significantly increased K-Ras (G12D) expression in NSCLC cells. Indicated cells were transfected with K-Ras (G12D) expression plasmid. After 72 hours of transfection, cells were subjected to Western blot analysis. **Figure S2** Expression of miR-199b in NSCLC cells that overexpressed or inhibited miR-199b. **a** H522 and H1975 cells were transfected with indicated negative oligonucleotides control (NC) or antisense nucleotides of miR-199 (ASO miR-199b). After 72 hours of transfection, cells were subjected to qRT-PCR analysis. **b** miR-199b was significantly decreased in stably expressing miR-199b antisense H522 cells. **c** A549 and H2122 cells were transfected with indicated negative oligonucleotides control (NC) or miR-199 mimics. After 72 hours of transfection, cells were subjected to qRT-PCR analysis. **d** miR-199b level was significantly increased in stably expressing miR-199b A549cells. **Figure S3** Aerosol delivery of miR-199b to the lung of mice. **a** Gene delivery efficiency of PCA_m_H_n_ as a gene carrier. Delivery efficiency of PCA_m_H_n_ as a gene carrier was evaluated using PCA_m_H_n_/green fluorescent protein (GFP) expression plasmid complex. ICR mice were exposed to aerosol containing PCA_m_H_n_/GFP expression plasmid complex or GFP expression plasmid only for 30 minuets, and 72 hours post-treatment, the mice were sacrificed for delivery efficiency assay. Green signals indicated that most of the delivered GFP was efficiently trasfected into lung. **b** miR-199b expression was measured in the lung tissue of K-Ras^LA1^ transgenic mice. Control mice were exposed to the gene carrier only (carrier); Vector group mice were exposed to vector mixed with the gene carrier (vector); miR-199b group mice were exposed to the miR-199b expression plasmid mixed with the gene carrier (miR-199b). **Figure S4** The protein levels of miR-199b candidate targets in NSCLC cells and the lungs of K-Ras^LA1^ transgenic mice. **a.** The expression levels of the indicated proteins in Fig. 4e were quantified using image J software. **b.** The expression levels of the indicated proteins in Fig. 4f were quantified using image J software. *,p<0.05 compare to carrier control; **, p<0.01 compared to carrier control; #, p<0.05 compared to vector control; ##, p<0.01 compared to carrier control. **Figure S5** The protein levels of Akt and ERK signaling pathway related genes in NSCLC cells. **a.** The expression levels of the indicated proteins in Fig. 5a were quantified using image J software. **b.** The expression levels of the indicated proteins in Fig. 5c were quantified using image J software. (PPTX 820 kb)

## Abbreviations

ERK: Extracellular regulated protein kinase
KSR2: Kinase suppressor of ras 2
NSCLC: Non-small cell lung cancer
PI3K: Phosphoinositide 3-kinase
PIK3R1: Phosphoinositide-3 kinase regulatory subunit 1
RHEB: Ras homolog enriched in brain
