# Supplementary material for: Restoration of mutant K-Ras repressed miR-199b inhibits K-Ras mutant non-small cell lung cancer progression
Source: J Exp Clin Cancer Res. 2019 Apr 15;38:165. doi: 10.1186/s13046-019-1170-7 (PMC6466664; doi:10.1186/s13046-019-1170-7)
Supplement: Supplementary file 1 — Table S1 Primer sequences used for qRT-PCR. Figure S1 Expression of K-Ras in NSCLC cells that overexpressed or silenced K-Ras. a shRNA of K-Ras significantly suppressed K-Ras expression in NSCLC cells. Indicated cells were transfected with indicated shRNA of K-Ras. After 72 hours of transfection, cells were subjected to Western blot analysis. b Transfection of K-Ras (G12D) expression vector significantly increased K-Ras (G12D) expression in NSCLC cells. Indicated cells were transfected with K-Ras (G12D) expression plasmid. After 72 hours of transfection, cells were subjected to Western blot analysis. Figure S2 Expression of miR-199b in NSCLC cells that overexpressed or inhibited miR-199b. a H522 and H1975 cells were transfected with indicated negative oligonucleotides control (NC) or antisense nucleotides of miR-199 (ASO miR-199b). After 72 hours of transfection, cells were subjected to qRT-PCR analysis. b miR-199b was significantly decreased in stably expressing miR-199b antisense H522 cells. c A549 and H2122 cells were transfected with indicated negative oligonucleotides control (NC) or miR-199 mimics. After 72 hours of transfection, cells were subjected to qRT-PCR analysis. d miR-199b level was significantly increased in stably expressing miR-199b A549cells. Figure S3 Aerosol delivery of miR-199b to the lung of mice. a Gene delivery efficiency of PCAmHn as a gene carrier. Delivery efficiency of PCAmHn as a gene carrier was evaluated using PCAmHn/green fluorescent protein (GFP) expression plasmid complex. ICR mice were exposed to aerosol containing PCAmHn/GFP expression plasmid complex or GFP expression plasmid only for 30 minuets, and 72 hours post-treatment, the mice were sacrificed for delivery efficiency assay. Green signals indicated that most of the delivered GFP was efficiently trasfected into lung. b miR-199b expression was measured in the lung tissue of K-RasLA1 transgenic mice. Control mice were exposed to the gene carrier only (carrier); Vector group mi [file 13046_2019_1170_MOESM1_ESM.docx]

**Table S1** Primer sequences. **Figure S1**  K-Ras expression in NSCLC cells. **a** K-Ras shRNA transfection suppressed K-Ras expression in NSCLC cells. **b** Transfection of K-Ras (G12D) expression vector increased K-Ras (G12D) expression in NSCLC cells. Indicated cells were transfected with indicated plasmids. After 72 hours of transfection, cells were subjected to Western blot analysis. **Figure S2**  miR-199b expression in NSCLC cells. **a** H522 and H1975 cells were transfected with negative oligonucleotides control (NC) or antisense nucleotides of miR-199 (ASO miR-199b). After 72 hours of transfection, cells were subjected to qRT-PCR analysis. **b** miR-199b was significantly decreased in stably expressing miR-199b antisense H522 cells. **c** A549 and H2122 cells were transfected with NC or miR-199 mimics. After 72 hours of transfection, cells were subjected to qRT-PCR analysis. **d** miR-199b level was significantly increased in stably expressing miR-199b A549cells. **Figure S3** Delivery efficiency of PCA_m_H_n_ as a gene carrier. **a** ICR mice were exposed to aerosol containing PCA_m_H_n_/GFP expression plasmid complex or GFP expression plasmid for 30 minuets, and 72 hours post-treatment, the mice were sacrificed for delivery efficiency assay. Green signals indicated that most of the delivered GFP was efficiently trasfected into lung. **b** miR-199b expression was measured in the lung tissue of K-Ras^LA1^ transgenic mice. Mice were exposed to the gene carrier only (carrier) or vector and gene carrier mixture (vector) or miR-199b expression plasmid and gene carrier mixture (miR-199b). **Figure S4** The expression levels of the indicated proteins in Fig. 4e (**a**) and Fig. 4f (**b**) were quantified using image J. *,p<0.05 compare to carrier control; **, p<0.01 compared to carrier control; #, p<0.05 compared to vector control; ##, p<0.01 compared to carrier control. **Figure S5**  The expression levels of the indicated proteins in Fig. 5a (**a**) and Fig. 5c (**b**) were quantified using image J.
